# Preoperative Glycemic Control Status as a Significant Predictor of Biochemical Recurrence in Prostate Cancer Patients after Radical Prostatectomy

**DOI:** 10.1371/journal.pone.0124761

**Published:** 2015-04-21

**Authors:** Hakmin Lee, Harim Kuk, Seok-Soo Byun, Sang Eun Lee, Sung Kyu Hong

**Affiliations:** Department of Urology, Seoul National University Bundang Hospital, Seongnam, Korea; National Health Research Institutes, TAIWAN

## Abstract

**Background:**

The effect of diabetes mellitus (DM) on prostate cancer (PCa) outcome remains controversial. Thus, we investigated the association of DM history, glycemic control, and metformin use with oncologic outcomes after radical prostatectomy (RP).

**Methods:**

We reviewed the records of 746 contemporary patients who had hemoglobin A1c (HbA1c) measured within the 6 months preceding RP. The associations between clinical variables and risk of adverse pathological features and biochemical recurrence (BCR) were tested using a multivariate logistic regression and multiple Cox-proportional hazards model, respectively. BCR was defined as prostatic specific antigen (PSA) > 0.2 ng/mL in 2 consecutive tests.

**Results:**

There were no significant differences in the rates of adverse pathologic features and BCR-free survival between patients with (n = 209) and without (n = 537) a history of DM diagnosis (all p > 0.05). In multivariate analyses, high HbA1c level (≥ 6.5%) was significantly related with high pathologic Gleason score (≥ 4+3; odds ratio [OR] 1.704, p = 0.019) and BCR-free survival (OR 1.853, p = 0.007). Metformin use was not associated with BCR-free survival (OR 0.662, p = 0.125).

**Conclusions:**

Poor glycemic control was significantly associated with BCR after RP. Meanwhile, metformin use was not associated with biochemical outcome after RP. Further investigation would be needed to identify exact mechanism underlying the impact of glycemic control on PCa treatment outcome.

## Introduction

The effect of diabetes mellitus (DM) on the risk and prognosis of prostate cancer (PCa) remains a controversial subject. DM has been linked to increased risks of various cancers [1]. Moreover, a meta-analysis found that cancer patients with DM had a significantly increased risk of mortality compared to cancer patients without DM [2]. However, unlike other cancers, published data have shown that the incidences of DM and PCa have an inverse relationship [2,3]. As for the outcome of PCa after primary treatment, some have reported no significant association with DM status, whereas others found significant associations in specific subsets of patients with PCa [4,5]. Overall, current literature suggests that the association between DM and PCa may not be simple.

Previously, we reported that a simple history of DM might not be a significant factor regarding clinico-pathologic features of PCa in men undergoing radical prostatectomy (RP) [6]. Conversely, in the same study, we observed that poor glycemic control, as represented by hemoglobin A1c (HbA1c) level, was significantly associated with adverse pathologic features, such as high Gleason score and extracapsular tumor extension, in men with DM who underwent RP for PCa. Subsequently, another group reported similar findings after analyzing data from the Shared Equal Access Regional Cancer Hospital (SEARCH) database [7]. Despite the observed association between preoperative HbA1c level and aggressive pathologic features of PCa in the two studies, data on the impact of glycemic control and the risk of biochemical recurrence (BCR) following RP are scarce. In the study utilizing the SEARCH database, a slightly increased risk of post-RP BCR was noted with higher HbA1c level, though statistically insignificant. Furthermore, increased risk of BCR was observed in certain subsets of diabetic men treated with primary radiotherapy for PCa [8]. Moreover, poor glycemic control has been shown to be associated with worse prognosis in other cancers [9,10]. Accordingly, it can be hypothesized that patients with poor glycemic control who are undergoing RP would have a worse prognosis. Thus, we investigated the clinical significance of glycemic control, represented by HbA1c level, relative to the postoperative biochemical outcome in men who underwent RP for clinically localized PCa.

## Patients and Methods

This study was approved by the Committee on the Ethics of the Seoul National University Bundang Hospital (Permit No. B1406/254-104). As a retrospective nature, there was no informed consent and all data were analyzed anonymously. We retrospectively reviewed records of 1,930 patients who underwent RP from January 2006 to June 2013 in a single tertiary center. Among all subjects, 760 patients who had an HbA1c measurement within 6 months before the surgery were identified. Many patients had PSA and HbA1c levels checked as a part of health check-up. If there were multiple HbA1c measurements within 6-months period before surgery, the mean value was regarded as being representative of preoperative glycemic control. After additional exclusion of 14 patients (other malignancy [n = 3], hormonal therapy [n = 8], radiotherapy [n = 3]), 746 subjects were included in our study. Preoperative characteristics such as biopsy result, prostate specific antigen (PSA) level, and clinical stage were collected by review of medical records. The history of DM was verified by self-administered questionnaires at admission, which asked if they had been diagnosed with DM during their lifetime. Only patients diagnosed with DM before surgery were considered to have a history of DM. Metformin use was also investigated at admission, and ≥3-month periods of metformin use were considered relevant. BCR was defined as PSA >0.2 ng/mL in two consecutive tests, which was performed at least 4 weeks after surgery. Pathologic outcomes such as seminal vesicle invasion, extraprostatic extension of tumor, positive surgical margin, and lymph node invasion were inspected as previously described [6]. High Gleason score was defined as ≥4 + 3. The follow-up time was defined as months from the date of surgery to the date of last visit or mortality.

Chi-square tests and independent t-tests were performed to evaluate differences between subgroups, and logistic regression tests were performed for univariate and multivariate analyses. Kaplan–Meier analyses evaluated differences in BCR according to DM history and HbA1c. The multiple Cox-proportional hazard model was used to analyze the association between various clinical variables and risk of BCR. All statistical analyses used an SPSS software package (SPSS 19.0, Chicago, IL, USA). A p-value <0.05 was considered statistically significant.

## Results

The patients’ characteristics are summarized in Table 1. Among all subjects, mean preoperative PSA was 12.0 ng/mL (range, 1.1–262 ng/mL), and 209 patients (28.0%) were identified to have a history of DM. Patients with HbA1c ≥ 6.5% and < 6.5% were considered to have “high” and “low” HbA1c, respectively. There were 192 patients (25.7%) in the high HbA1c group and 554 patients in the low HbA1c group. There was no significant difference in follow-up times between two groups (p = 0.063). Notably, among patients with high HbA1c, 56 patients (29.2%) had no previous history of DM. During median follow-up time of 43 months (IQR 22–64.3), 147 patients (19.7%) developed BCR. The median time from RP to BCR were 12 months (IQR 5–30) for entire patients, 14.0 months for low HbA1c group, and 9.5 months for high HbA1c group, respectively.

**Table 1:** Patients’ characteristics.

N = 746	Mean (range) or Counts (percent of total)
Age (years)	66.3 (39–82)
BMI (kg/m^2^)	24.4 (21–33.6)
PSA (ng/mL)	12.0 (1.1–262)
Prostate volume (g)	38.1 (9.5–141.0)
History of DM	
Positive	209 (28.0%)
Negative	537 (72.0%)
HbA1c	
≥6.5 (%)	192 (25.7%)
<6.5 (%)	554 (74.3%)
Metformin user	135 (18.1%)
Biopsy Gleason score	
≤6	364 (48.8%)
7	260 (34.9%)
≥8	122 (16.4%)
Clinical stage	
T1	467 (62.6%)
≥T2	279 (37.4%)
Pathologic Gleason score	
≤3+4	365(49.0%)
≥4+3	381 (51.0%)
Pathologic stage	
T2	506 (67.8%)
T3a	169 (22.7%)
≥T3b	71 (9.5%)
Surgical approach	
Open	287 (38.5%)
Laparoscopic	13 (1.7%)
Robotic	446 (59.8%)
Positive surgical margin	213 (28.6%)
Seminal vesicle invasion	66 (8.8%)
Extraprostatic extension	235 (31.5%)
Lymph node invasion	25 (3.3%)

BMI = body mass index; PSA = prostatic specific antigen; DM = diabetes mellitus; HbA1c = hemoglobin A1c

In Table 2, the distribution of clinico-pathologic characteristics was compared between subgroups, which were divided by DM history or HbA1c level. The high HbA1c group showed significantly less favorable clinico-pathologic characteristics compared with the low HbA1c group. Preoperative PSA (p = 0.001), biopsy Gleason score (p < 0.001), pathologic stage (p = 0.002), pathologic Gleason score (p = 0.008) and rates of BCR (p < 0.001) were all significantly higher in high HbA1c groups. Similarly, adverse pathologic findings such as positive surgical margin (p = 0.002), seminal vesicle invasion (p = 0.001), extraprostatic extension (p < 0.001), and lymph node invasion (p = 0.010) were more prevalent in the high HbA1c group. When we compared diabetic versus non-diabetic (defined by DM history), there were no significant differences in clinico-pathologic findings including rate of BCR.

**Table 2:** Differences in clinico-pathologic findings according to DM history and HbA1c level.

	DM positive	DM negative	p-value	HbA1c ≥6.5%	HbA1c <6.5%	p-value
Number of patients	209	537		192	554	
Mean age (years)	66.9	66.1	p = 0.226	66.7	66.2	p = 0.236
Mean PSA (ng/mL)	12.9	11.6	p = 0.284	14.7	11.1	p = 0.001
Mean BMI (kg/m^2^)	24.5	24.3	p = 0.215	24.8	24.2	p = 0.063
Mean PV (g)	37.5	37.8	p = 0.225	37.7	37.8	p = 0.727
Biopsy Gleason score						
≤6	89(42.6%)	275(51.2%)	p = 0.101	70(36.5%)	294(53.1%)	p<0.001
7	83(39.7%)	177(33.0%)		83(43.2%)	177(31.9%)	
≥8	37(17.7%)	85(15.8%)		39(20.3%)	83(15.0%)	
Clinical Stage						
T1	127(60.8%)	340(63.3%)	p = 0.761	114(59.4%)	353(63.7%)	p = 0.162
≥T2	82(39.2%)	197(36.7%)		78(40.6%)	201(36.3%)	
Pathologic Gleason score						
≤3+4	93(44.5%)	272(50.7%)	p = 0.142	79(41.1%)	286(51.6%)	p = 0.008
≥4+3	116(55.5%)	265(49.3%)		113(58.9%)	268(48.4%)	
Pathologic Stage						
T2	143(68.4%)	363(67.6%)	p = 0.253	112(58.3%)	394(71.1%)	p = 0.002
T3	66(31.6%)	167(31.1%)		79(41.1%)	154(27.8%)	
T4	0(0.0%)	7(1.3%)		1(0.5%)	6(1.1%)	
PSM	60(28.7%)	153(28.5%)	p = 0.510	71(37.0%)	142(25.6%)	p = 0.002
SVI	15(7.2%)	51(9.5%)	p = 0.197	27(16.7%)	39(7.0%)	p = 0.001
EPE	65(31.1%)	170(31.7%)	p = 0.478	79(41.1%)	156(28.2%)	p<0.001
LNI	9(4.3%)	16(3.0%)	P = 0.366	12(6.3%)	13(2.3%)	P = 0.010
BCR	43(20.6%)	104(19.4%)	p = 0.759	56(29.2%)	91(16.4%)	p<0.001

DM = diabetes mellitus; HbA1c = hemoglobin A1c; PSA = prostate specific antigen; BMI = body mass index; PV = prostate volume; PSM = positive surgical margin; SVI = seminal vesicle invasion; EPE = extraprostatic extension; LNI = lymph node invasion; BCR = biochemical recurrence.

Multivariate logistic regression analyses were used to determine if HbA1c level was significantly related to several clinico-pathologic outcomes (Table 3). Univariate analysis revealed that a high HbA1c level was significantly associated with each adverse pathologic finding, including pathologic Gleason score (≥ 4+3), pathologic stage (≥ T3), extracapsular extension, seminal vesicle invasion, positive surgical margin, and lymph node invasion. However, only pathologic Gleason score (≥ 4+3) reached statistical significance in multivariate analysis adjusted for age, PSA, body mass index, biopsy Gleason score, and clinical stage (odds ratio 1.704, p = 0.019).

**Table 3:** Multivariate analyses of the impact of high preoperative HbA1c level (≥6.5%) on various pathologic outcomes after radical prostatectomy.

	Univariate analysis			Multivariate analysis		
	OR	95% CI of OR	p-value	OR	95% CI of OR	p-value
Pathologic Gleason score (≥4+3)	2.060	1.474–2.879	<0.001	1.704	1.093–2.656	0.019
Pathologic Stage (≥T3)	1.759	1.251–2.473	0.001	1.336	0.892–2.001	0.160
Extracapsular extension	1.784	1.267–2.511	0.010	1.372	0.914–2.059	0.127
Seminal vesicle invasion	2.161	1.283–3.638	0.004	1.544	0.814–2.930	0.184
Positive surgical margin	1.702	1.200–2.415	0.003	1.408	0.962–2.060	0.078
Lymph node invasion	2.774	1.243–6.190	0.013	2.039	0.818–5.080	0.126

Multivariate analyses were adjusted for age, prostatic specific antigen, body mass index, biopsy Gleason score, and clinical stage

HbA1c = hemoglobin A1c; OR = odds ratio; CI = confidence interval.

**Fig 1:**
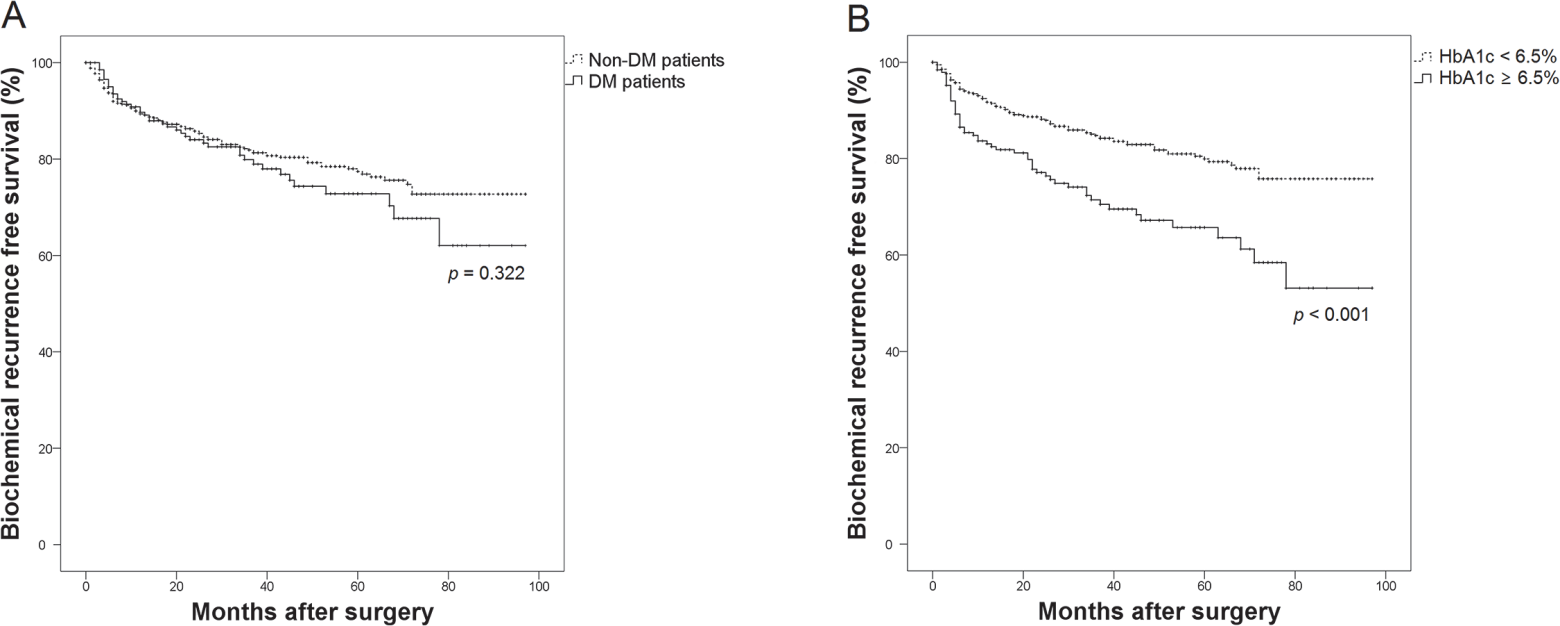
Kaplan-Meier curves for biochemical recurrence-free survival according to: (A) the history of DM; (B) HbA1c level. DM = diabetes mellitus, HbA1c = hemoglobin A1c

A Kaplan–Meier analysis showed a significant difference in BCR-free survival according to HbA1c level (Fig 1). Although no significant difference was observed according to simple history of DM, the high HbA1c group showed poor prognosis in terms of BCR compared with the low HbA1c group (p < 0.001). From the Cox-proportional hazards analysis, the variables of PSA level, pathologic Gleason score, pathologic stage, margin status and high HbA1c level were revealed as significant prognostic factors of BCR after RP (Table 4). Meanwhile, metformin use was not observed to have a significant influence on BCR.

**Table 4:** Univariate and multivariate Cox proportional hazards analyses for potential predictors of biochemical recurrence after radical prostatectomy.

	Univariate analysis			Multivariate analysis		
	OR	95% CI of OR	p-value	OR	95% CI of OR	p-value
Age	1.030	1.003–1.058	0.030	1.001	0.972–1.031	0.939
BMI	1.037	0.976–1.103	0.241	1.034	0.963–1.110	0.361
PSA	1.025	1.021–1.029	<0.001	1.008	1.002–1.015	0.012
PV	0.997	0.986–1.008	0.584	0.993	0.981–1.006	0.284
HbA1c ≥ 6.5%	2.047	1.467–2.857	<0.001	1.853	1.187–2.893	0.007
Pathologic						
GS≤6	1.00	(ref)		1.00	(ref)	
GS = 7	12.739	3.126–51.911	<0.001	4.830	1.159–20.126	0.031
GS≥8	76.310	18.568–313.619	<0.001	19.452	4.538–83.373	<0.001
Pathologic stage						
T2	1.00	(ref)		1.00	(ref)	
T3	9.743	6.539–14.517	<0.001	4.051	2.565–6.399	<0.001
T4	12.495	4.582–32.174	<0.001	2.360	0.851–6.549	0.099
PSM	5.723	4.050–8.086	<0.001	1.887	1.270–2.803	0.002
LNI	6.746	4.096–11.113	<0.001	0.652	0.384–1.109	0.505
Metformin	1.293	0.870–1.922	0.204	0.662	0.390–1.122	0.125

OR = odds ratio; CI = confidence interval; BMI = body mass index; PSA = prostate specific antigen; PV = prostate volume; HbA1c = hemoglobin A1c; GS = Gleason score; PSM = positive surgical margin; LNI = lymph node invasion

## Discussion

We observed that a simple history of DM was not associated with aggressive pathologic features or postoperative biochemical outcome in patients undergoing RP. However, the level of preoperative glycemic control, as represented by HbA1c level, was significantly associated with high pathologic Gleason score and postoperative BCR-free survival regardless of previous DM history. Such findings suggest that glycemic control, rather than simple history of DM, is a more significant factor for oncological outcome following RP. Accordingly, our results may explain inconsistent results of previously published data about the impact of DM on post-RP outcomes [11–15]. In contrast, metformin use was not a significant predictor of postoperative biochemical outcome in our study.

Previously, other groups looked at the potential association between DM and PCa outcome after RP, reporting variable findings. Utilizing the CaPSURE database, Chan et al. reported that DM was not associated with BCR after RP [11]. However, Patel et al. reported that DM was significantly associated with an increased likelihood of BCR after RP [12]. In their study, 5-year postoperative BCR-free survival was 75% for the non-DM group, compared with 62.5% for the DM group. Conversely, Rieken et al. evaluated 6,863 patients who underwent RP and failed to detect a significant association between DM and increased BCR [13]. Yet, a study of an RP cohort included in the Shared Equal-Access Regional Cancer Hospital database showed that DM was associated with a 2.5-fold increased risk of BCR only among white obese men, whereas DM was not significantly associated with BCR among the overall cohort [14]. Similarly, Wu et al. observed that DM was a significant predictor of metastasis only for obese men while not being a risk factor for progression to metastases among the whole RP cohort [15]. Here, we also found no association between DM and BCR following RP. Accordingly, most reported data suggests that, overall, simple history of DM is not associated with disease progression or recurrence after RP.

Not many have reported on the impact of glycemic control or glucose regulation, independent of DM diagnosis, on the outcome of PCa. From the analysis of post-load plasma glucose concentration, Gapstur et al. reported that hyperglycemia was significantly associated with PCa mortality [16]. Although only patients with known DM were analyzed, a previous study from our group revealed that preoperative HbA1c level was significantly associated with higher pathologic Gleason score after RP [7]. Analyzing serum glucose level at the time of treatment and the risk of PCa recurrence in 1,734 men treated with RP or radiation therapy (RT), Wright et al. showed that men with elevated glucose (≥100 mg/dL) had a 50% increased risk of recurrence [17]. In addition, Ma et al. found that men in the highest C-peptide (an insulin surrogate) quartile had a >2-fold increased risk of PCa-specific mortality than those in the lowest C-peptide quartile [18]. Despite the application of various surrogate markers, overall, the results from all these studies support our finding that poor glycemic control or glucose regulation is an independent predictor of disease recurrence among men undergoing RP. Moreover, compared with a single serum glucose test, HbA1c level would be a more informative measure of a patients’ glycemic control status.

Considering discrepancies between our current findings and those from other studies regarding DM and the outcome of PCa, it can be deduced that a simple diagnosis of DM may not reflect an individual’s current or recent level of glycemic control. It should be recalled that men with well-controlled DM might have HbA1c levels comparable to those without DM. Moreover, because a proportion of the adult general population is thought to have undiagnosed DM, exclusion of men with no history of DM may not have been an appropriate approach for studying the impact of glycemic control on PCa outcome. Accordingly, the effect of glycemic control on biochemical outcome may have been underestimated in studies on men with DM only.

Studies on other cancers also found glucose regulation to be clinically significant. In a study of 202 women with breast cancer, a trend toward increased risk of recurrence was noted with increasing serum glucose level [19]. Another study on colorectal cancer patients demonstrated that poorly controlled DM, as judged by HbA1c level, independently predicted a more advanced disease and poorer 5-year survival [9]. Moreover, poor glycemic control (HbA1c ≥6.5%) was independently associated with postoperative tumor recurrence after curative resection in diabetics with hepatocellular carcinoma [10]. These reports provide further evidence that abnormal glucose regulation may affect cancer outcome.

Currently, obesity is widely regarded as increasing the risk of PCa-specific mortality [18]. As impaired glucose regulation or DM commonly accompanies obesity, alterations in glucose metabolism may likely contribute to PCa progression or mortality. Several hypotheses can be suggested on the underlying mechanism for the impact of glycemic control on PCa outcome. Cancer cells use more glucose than normal cells, and hyperglycemia elicits hyperinsulinemia and activation of insulin/insulin-like growth factor pathway, which has been linked to aggressive PCa [1]. In addition, poor glycemic control may lead to production of advanced glycosylated end products, which may initiate lipid peroxidation and produce genotoxic aldehyde, resulting in DNA damage [9]. In addition, poor glycemic control can inhibit cellular ascorbic acid uptake, which may cause immunosuppression. Such effects of hyperglycemia have been mentioned as contributing to disease progression in cancer patients. Furthermore, chronic inflammation accompanying DM and metabolic syndrome results in the release of cytokines that can promote cancer growth [20]. Alternatively, the relationship between HbA1c and testosterone may also contribute to causality. Several studies reported that low preoperative testosterone is related to worse prognostic factors, such as higher Gleason score, pathologic stage, and PSA level, and to higher risk of BCR [21,22]. Meanwhile, others demonstrated that poor glycemic controls is related to lower testosterone levels [23]. Thus, one can infer that high HbA1c can be related to worse prognosis of PCa.

Both diabetes and PCa are highly prevalent diseases with increasing incidence [1]. PCa is the second most common cancer in men and the sixth leading cause of death among all cancer cases worldwide. In 2008, 914,000 individuals were diagnosed with PCa, and 258,000 men died of PCa worldwide [24]. Besides, 285 million people are estimated to have type 2 DM globally, with apparent correlation with age and obesity [1]. In 2011, the prevalence of DM has been reported to reach 26.9% among American adults 65 years or older [25]. These epidemiologic findings emphasize the clinical value of present study.

Metformin medication was not found to be a significant predictor of BCR-free survival in the present study, even when we constrained metformin medication to longer period (>6months) (HR 0.666, p = 0.133). Similarly, Patel et al. reported that metformin use had no significant benefit among men undergoing RP [12]. Others also failed to reveal beneficial effects of metformin in patients with PCa after RP [13]. However, Spratt et al. showed that metformin might improve outcome after external-beam RT and reduce development of castration-resistant PCa [8]. Despite only 157 of 2,901 subjects included in their study using metformin, it apparently improved BCR-free survival, distant metastasis-free survival, PCa-specific survival, and overall survival. Such findings suggest that synergism might exist between metformin and radiation because both affect the AMP kinase pathway and lead to decreased cell growth and cell cycle progression. Preclinical investigations showed that metformin can inhibit the growth of numerous cancer cell lines including those from PCa [26]. Studies on patients with advanced PCa demonstrated that metformin provided overall survival benefits [27]. Still, some have argued that this may be due to cardio-protective rather than anti-cancer effects [13]. As controversy continues about the therapeutic benefit of metformin in cancer patients, further investigations are warranted, preferably randomized, prospective studies.

Our study may be limited by its retrospective design and the relatively small number of subjects compared to other RP surveys. Not all men undergoing RP had HbA1c measured preoperatively, and we could not assess the exact duration of DM. Further, despite assessing metformin use, the effects of other drugs could not be included in our analyses. Our study population was limited to patients with RP, and comorbidities other than DM were not accounted for. Moreover, more meaningful outcomes, such as metastasis-free or PCa-specific survival, could not be assessed due to lack of long-term follow-up. However, to our knowledge, our study is the first to reveal the clinical significance of glycemic control, as represented by HbA1c level, on the clinical and oncological outcomes in men undergoing RP.

## Conclusions

Our study showed that glycemic control status, as represented by HbA1c level, was an independent predictor of biochemical outcome after RP. On the other hand, Metformin use did not have a significant effect on biochemical outcome after RP. Further evaluation would be needed to identify the mechanism underlying the impact of glycemic control on the outcome of PCa.
